# New Insights into the Modification of the Non-Core Metabolic Pathway of Steroids in *Mycolicibacterium* and the Application of Fermentation Biotechnology in C-19 Steroid Production

**DOI:** 10.3390/ijms24065236

**Published:** 2023-03-09

**Authors:** Yang Zhang, Peiyao Xiao, Delong Pan, Xiuling Zhou

**Affiliations:** School of Life Science, Liaocheng University, Liaocheng 252000, China

**Keywords:** C-19 steroids, *Mycolicibacterium*, non-core metabolic pathway of steroids, propionyl-CoA, fermentation biotechnology

## Abstract

Androsta-4-ene-3,17-dione (AD), androsta-1,4-diene-3,17-dione (ADD), and 9α-hydroxy-4-androstene-3,17-dione (9-OHAD), which belong to C-19 steroids, are critical steroid-based drug intermediates. The biotransformation of phytosterols into C-19 steroids by *Mycolicibacterium* cell factories is the core step in the synthesis of steroid-based drugs. The production performance of engineered mycolicibacterial strains has been effectively enhanced by sterol core metabolic modification. In recent years, research on the non-core metabolic pathway of steroids (NCMS) in mycolicibacterial strains has made significant progress. This review discusses the molecular mechanisms and metabolic modifications of NCMS for accelerating sterol uptake, regulating coenzyme I balance, promoting propionyl-CoA metabolism, reducing reactive oxygen species, and regulating energy metabolism. In addition, the recent applications of biotechnology in steroid intermediate production are summarized and compared, and the future development trend of NCMS research is discussed. This review provides powerful theoretical support for metabolic regulation in the biotransformation of phytosterols.

## 1. Introduction

**Figure 1:**
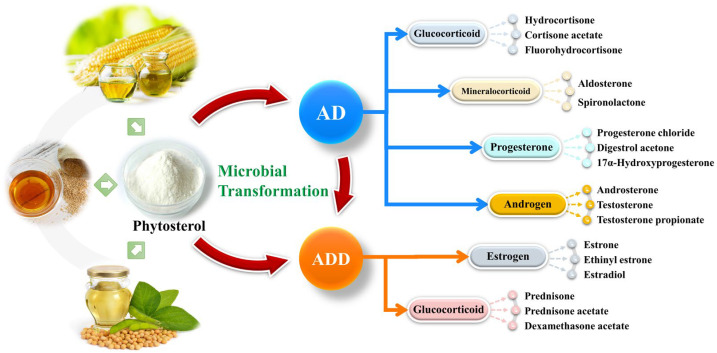
AD produced by the microbial transformation of phytosterol can be used as the starting molecule to synthesize most steroid drugs.

Steroids are cyclic terpenoid lipids with various complex structures. The molecular structure of steroids has a steroid nucleus composed of a five-membered ring and three six-membered rings. Steroids have different functions depending on their substituents, double bond positions, or stereoconfiguration in the nucleus. Steroids are widely present in animals, plants, and microorganisms and play important bioactive functions [1]. In the clinic, steroidal hormone drugs have obvious physiological and pharmacological functions, such as anti-infection, anti-allergy, and antitumor activities, which are just-needed medications [2,3]. The biosynthesis of steroid pharmaceutical intermediates from phytosterols using mycolicibacterial strains, especially *Mycolicibacterium* sp. (basonym: *Mycobacterium* sp.) and *Mycolicibacterium neoaurum* (previously known as *Mycobacterium neoaurum*), is the current well-established steroidal industrial route. The C-19 steroid pharmaceutical intermediates produced by *Mycolicibacterium* mainly include androst-4-en-3,17-dione (AD), androsta-1,4-diene-3,17-dione (ADD), 9α-hydroxyandrostra-4-ene-3,17-dione (9OH-AD), and 4-androstene-17β-ol-3-one (TS). AD can be used to produce various steroid derivatives, such as cortisone, spironolactone, progesterone, testosterone, progesterone, and prednisone [4]. AD can also be converted to ADD in one step by 3-ketosteroid-∆1-dehydrogenase (KstD), which is a precursor for estrogens and glucocorticoids [5]. Thus, almost all steroid drugs can be synthesized from AD (Figure 1) [6]. The establishment of a technology system for the microbial synthesis of AD with phytosterol as a substrate has now become the main production method of hormone steroid drugs [1]. Promoting the green, efficient, and low-cost production of AD has become a major strategic demand of the global steroid drug industry.

As early as the 1940s, microorganisms were found to have the ability to degrade cholesterol [4]. Strains of the family *Mycobacteriaceae* have powerful sterol-degradation systems and mycolic acids in cell walls promote steroid uptake. These are ideal strains for the production of intermediate for steroid drug synthesis [7]. After mutagenesis, some strains of the genus *Mycolicibacterium* (basonym: *Mycobacterium*), especially *M. neoaurum*, become important strains for industrial AD production [3]. Benefiting from the rapid development of biotechnology, multiple steroid drug intermediates production strains have undergone genomic, transcriptomic, and other omics analyses. The sterol metabolic pathway has been analyzed in detail through the identification of intermediate products and the analysis of omics information, and the metabolic pathway of sterol has been analyzed in detail [8,9]. Metabolic engineering methods have been increasingly used to construct and optimize strains that accumulate steroid intermediates [1]. In the past five years, the research of the non-core metabolic pathway of steroids (NCMS) based on cofactor, transcription factor, and substrate transport engineering has gradually attracted attention. Many strains with good industrial application potential have been developed in the NCMS research field. Furthermore, applying various biotechnologies, such as fermentation and transformation, will greatly promote the development of the steroid drug industry [10,11,12,13,14,15,16]. This article focuses on the modification of the NCMS in *Mycolicibacterium* and the application of biotechnology in synthesizing C-19 steroid drug intermediates over the last five years.

## 2. Core Metabolic Pathway of Steroid (CMS) in *M. neoaurum*

**Figure 2:**
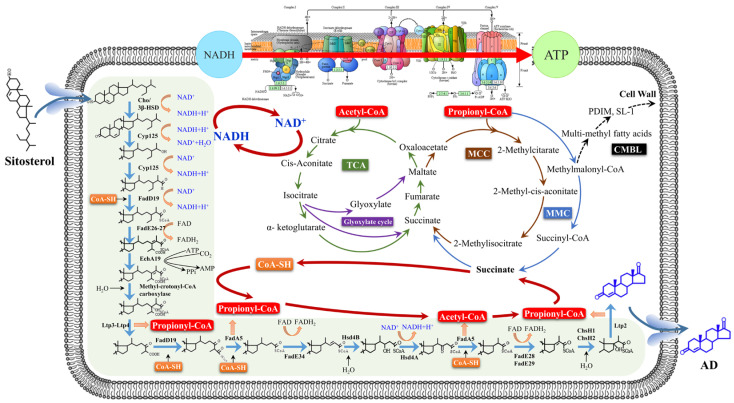
Schematic map of the metabolic pathway of β-sitosterol to AD by mycolicibacterial strains. The shaded green part is the core metabolic pathway. The Oxidative phosphorylation diagram is from https://www.kegg.jp/pathway/mne00190 (accessed on 12 January 2023).

The genus *Mycobacterium* is currently divided into an emended genus *Mycobacterium* and four novel genera, *Mycolicibacterium*, *Mycolicibacter*, *Mycolicibacillus*, and *Mycobacteroides*, according to the comprehensive phylogenomic analyses and numerous identified molecular signatures [17,18]. Most of the strains with C-19 steroid production belonged to the *Mycolicibacterium* genus. Studies on the mycolicibacterial strains steroid metabolic pathway usually focus on *M. neoaurum* and *Mycolicibacterium smegmatis* (basonym: *Mycobacterium smegmatis*). Many species of microorganisms have been proven to have steroid drug intermediate production capacity; for example, *Aspergillus oryzae* and *Fusarium moniliforme* are able to produce AD with sterols as substrates [19,20] and *Chryseobacterium gleum* and *Gordonia neofelifaecis* are able to use cholesterol to produce ADD [21,22]. With continuous progress in strain mutagenesis selection technology, *Arthrobacter*, *Brevibacterium*, *Pseudomonas*, and *Rhodococcus* have shown AD production capacity, but only mycolicibacterial strains have been successfully used in industrial-scale production [23]. From omics studies, such as genomes, transcriptomes, and the identification of intermediates, the gene clusters for steroid utilization by a variety of microorganisms have been localized and steroid degradation pathways have been established. As reviewed by Zhao et al. (2021) [1], steroid metabolism in mycolicibacterial strains includes three main processes: steroid uptake and transport, steroid side chain degradation, and the complete oxidation of the steroid nucleus. To achieve the maximum accumulation of steroid drug intermediates, production strains have lost the ability to degrade the steroid nucleus through mutagenesis screening or genetic modification. Therefore, the content related to steroid nucleus degradation is not beyond the scope of this review. Based on the literature, taking the metabolism of β-sitosterol to AD by *M. neoaurum* as an example, the core metabolic pathway and related metabolism of steroids were reviewed (Figure 2).

The metabolic process of AD production by *M. neoaurum* is mainly the phytosterol-side chain degradation process. The degradation process is similar to the degradation process of fatty acid β-oxidation. The Phytosterols must be activated at the beginning of metabolism. The key enzymes involved in activation are cholesterol oxidase (ChO) and 3β-hydroxysteroid dehydrogenase (3β-HSD), which play different dominant roles in different microorganisms. Yao et al. (2013) [24] identified two ChO isozymes, ChoM1 and ChoM2, of which ChoM2 plays a key role in microbial uptake and sterol metabolism. In contrast, 3β-HSD is a key enzyme for sterol parent nucleus oxidation in strains such as *M. smegmatis* and *Comamonas testosteroni* [25,26]. In addition, most of the existing studies support that, the initial stage of oxidation of the 3beta-hydroxy group in a ring A of the steroid nucleus is catalyzed by 3β-HSD [27,28]. The cholesterol-side chain is activated by the steroid C27 monooxygenase (SMO), such as Cyp125, which are able to hydroxylate substrates and further oxidize them to form carboxylic acids. Sterols can only be esterified by acyl-CoA ligase (FadD) when their side chains form carboxylic acids. Carboxyl-CoA produced by esterification undergoes multiple rounds of β-oxidation-like processes to be completely degraded [29]. Depending on whether the product of steroid degradation is AD or 22-hydroxy-23,24-bisnorchol-4-ene-3-one (4-HBC), degradation can be divided into AD and HBC subpathways [1]. In the AD subpathway, 24-ethyl-3-oxo-4-cholestene-27-oic acid (27-EOCS) is activated by FadD19 and catalyzed by acyl-CoA dehydrogenase, enoyl-CoA hydratase, hydroxyacyl-CoA dehydrogenase, and thiolase. The β-oxidation-like process involves the acetyl-CoA thiolase (FadA) family, the β-hydroxyacyl-CoA dehydrogenase (FadB) family, the fatty-acid-CoA ligase (FadD) family, the acyl-CoA dehydrogenase (FadE) family, and the EchA family (enoyl-CoA hydratase). Unlike cholesterol, β-sitosterol contains an ethyl branch chain in its side chain. Thus, after the first round of dehydrogenation, carboxylation, rather than hydration, occurs at the C28 site, which is catalyzed by methylcrotonyl-CoA carboxylase [30]. In the AD subpathway, the first two cycles of β-oxidation produce two molecules of propionyl-CoA and one molecule of acetyl-CoA. In the third round of β-oxidation, another propionyl-CoA is released in a retro-aldol manner in the last cycle [31].

## 3. Non-Core Metabolic Pathway of Steroids in *M. neoaurum*

### 3.1. Phytosterol Uptake

The uptake and transport of sterols in *M. neoaurum* are mainly mediated by the Mce 4 transporter in the mammalian cell entry (Mce). Mce4 is an ABC transporter that plays an important role in sterol uptake by *Mycolicibacterium* [1,32]. The Mce4 transporter system comprises many proteins with different functions. Six Mce proteins (Mce4ABCDEF) are located on the cell wall: two membrane proteins (Mam4A and Mam4B), two permeases (YrbEA and YrbEB), one ABC transporter ATPase (MceG), and two orphaned Mce-associated membrane proteins [33]. It has been proposed that OmamA and LucA stabilize the function of the Mce4 system and play a role in cholesterol and fatty acid uptake [34,35]. Mce4 deletion affects the uptake of sterols by mycolicibacterial strains as well as the growth and morphology of sterols [36].

In addition, sterol uptake also depends on direct contact between the cell envelope and sterol particles. The core of the *Mycolicibacterium* cell wall is a complex formed through the covalent attachment of mycolyl-arabinogalactan-peptidoglycan (m-AG-PG) [37]. Lipid capsules, including trehalose monomycolate (TMM), trehalose dimycolate (TDM), acyl lipids, and proteins, are attached to the covalent layer [38,39]. The acyl lipids mainly includes phthiocerol dimycocerates (PDIM), polytrehalose, diacyltrehalose (PAT/DAT), and sulfoglycolipid-1 (SL-1), synthesized from propionyl-CoA and acetyl-CoA [40,41]. The thickness of the cell wall and the attachment layer will affect the sterol uptake. When additional inhibitors (such as vancomycin or glycine, etc.) that can hinder cell envelope synthesis are added, the efficiency of sterol uptake by cells is significantly accelerated, and the yield of the corresponding products is also significantly increased [42]. In addition, the deletion of the mycolic acid synthetase, such as the arabinofuranosyl transferase encoded by *embC* gene, blocks cell wall synthesis [43]. The antigen 85 (Ag85) complex encoded by *fbpA*, *fbpB*, and *fbpC* was the key factor for producing mycolylarabinogalactan (m-AG) and trehalose dimycolate (TDM) in the mycolicibacterial strains cell envelope [44,45]. The inhibition of Ag85C was able to block the biosynthesis of the cord factor TDM in *M. tuberculosis* [46]. Therefore, genes related to the cell wall and attachment layer synthesis are potential targets for improving sterol uptake.

### 3.2. Phytosterol-Side Chain Degradation and Coenzyme I Metabolism

Phytosterol side-chain degradation by *Mycolicibacterium* involves multiple cofactors and the formation of many high-energy compounds. In addition to NADH and FADH_2_, propionyl-CoA and acetyl-CoA are produced in large quantities [47,48]. These classes of Coenzyme A substances containing high-energy thioester bonds produce eighteen molecules of NADH and seven molecules of FADH_2_ after metabolism. When a molecule of β-sitosterol is converted into AD, 80 molecules of ATP are generated through the electron transport chain (ETC) [30]. To produce AD from β-sitosterol, the cofactor change formula was β-sitosterol + 21H_2_O + 4ATP + 7GDP + 7Pi + 10FAD + 21NAD^+^ = AD (D) + 21/2CO_2_ + 4AMP + 4PPi + 7GTP + 10FADH_2_ + 21NADH + 21H^+^. In this process, four molecules of ATP are consumed and ten molecules of FADH2 and twenty-one molecules of NADH are produced. Under the action of ETC and oxygen, the reduced coenzyme produces an oxidative coenzyme, which makes the cofactors circulate effectively. Therefore, in AD synthesis, the content and ratio of NAD^+^ and NADH are important factors affecting phytosterol transformation.

### 3.3. Phytosterol-Side Chain Degradation and Propionyl-CoA Metabolism

The microbial intracellular coenzyme A library is mainly composed of coenzyme A derivatives, such as acetyl-CoA, propionyl-CoA, and succinyl-CoA [49]. They participate in various intracellular syntheses and catabolism and have regulatory roles in certain key enzymes [50]. The transformation of phytosterol into AD by *Mycolicibacterium* produces large amounts of propionyl-CoA and acetyl-CoA [51]. Enhancing sterol-side chain metabolism efficiency means producing more coenzyme A substances, such as propionyl-CoA and acetyl-CoA. However, the excessive intracellular accumulation of propionyl-CoA is toxic to *Mycolicibacterium* [52].

Three pathways are involved in the metabolism of propionyl-CoA in mycolicibacterial strains for detoxification (Figure 2). They are the 2-methylcitrate cycle pathway (MCC), the methyl-branched polyketide lipid synthesis pathway (MBPL), and the methylmalonyl cycle pathway (MMC) [53]. In MMC, propionyl-CoA is first carboxylated into (S)-methylmalonyl-CoA, then racemized into the (R)-enantiomer, and finally isomerized into succinyl-CoA, which enters the tricarboxylic acid cycle (TCA). This metabolic process is catalyzed by propionyl-CoA carboxylase (PCC), methylmalonyl-CoA epimerase (MCEE), and methylmalonyl-CoA mutase (MTU) [54]. The second pathway is MBPL, in which propionyl-CoA is converted into (S)-methylmalonyl-CoA, which can contribute to the biosynthesis of cell wall lipids, such as phthiocerol dimycocerosates (PDIM) or sulfolipids (SL-1) [55]. MCC is the third metabolic detoxification pathway of propionyl-CoA in *Mycolicibacterium* that transforms propionyl-CoA into succinic acid [56]. In the MCC pathway, propionyl-CoA condenses with oxaloacetic acid under the action of methyl citrate synthase (MCS or PrpC, encoded by *prpC*) to produce 2-methylcitric acid (2-MC). 2-MC is subsequently dehydrated by methyl citrate dehydratase/hydratase (PrpD; encoded by *prpD*) or a combination of methyl citrate dehydratase (AcnD) and methyl aconitrate cis-trans isomerase (PrpF) to produce methyl aconitate. Subsequently, methyl aconitrate is rehydrated by aconitase (AcnB) to form methyl isocitrate. Methyl isocitrate is cleaved by methyl isocitrate lyase (MCL or PrpB, encoded by *prpB*) or isocitrate lyase to produce pyruvate and succinate [57]. Eventually, succinate and succinyl-CoA produced by the MCC and MMC pathways are converted into TCA by succinic dehydrogenase (sdhABCD) and succinyl-CoA synthase (sucBCD), respectively, to generate energy. Therefore, the metabolism of propionyl-CoA plays an essential role in maintaining energy supply and preventing the excessive accumulation of propionyl-CoA during the metabolism of sterols and odd-chain fatty acids by *Mycolicibacterium* [54].

### 3.4. Phytosterol-Side Chain Degradation and Reactive Oxygen Species

Reactive oxygen species (ROS), including hydrogen peroxide (H_2_O_2_), superoxide anion (O_2_^−^), and hydroxyl radical (OH^−^), are chemically active oxidation molecules produced during aerobic metabolism in organisms. When intracellular ROS accumulate to a certain level, excessive ROS lead to oxidative stress on cell metabolism, destroying various biomolecules, such as DNA, lipids, and proteins, and ultimately leading to cell damage, which reduces the fermentation performance of the strains [58,59]. The degradation of phytosterol-side chain in mycolicibacterial strains produces a large amount of NADH, and the majority of NADH is converted into NAD^+^ by NADH dehydrogenase on the ETC. NADH dehydrogenase on the ETC can be divided into three categories: type I NADH dehydrogenase (NADH: Q oxidoreductase; NDH-I); type II NADH dehydrogenase (NDH-II); and sodium ion-driven NADH dehydrogenase (Nqr) [60]. NDH-I is the main producer of reactive oxygen species (ROS) in the ETC, especially when the concentration of succinic acid is high, the concentration of oxaloacetic acid or malic acid is low, and the ratio of NADH/NAD^+^ is increased. An increase in ROS can also induce oxidative stress, leading to cell decay [61]. Nqr exists only in prokaryotes, and it may only participate in the sodium ion balance in this type of bacteria [62]. NDH-II is widely found in the bacteria and mitochondria of some fungi, plants, and protozoa. Compared to NDH-I, the amount of ROS produced by NDH-II was very weak. Therefore, the electron transfer initiated by NDH-II is able to reduce the damage caused by strong oxidizing free radicals to cells [63].

The metabolism of sterols involves many oxidoreductases, such as Cho and acyl-CoA dehydrogenases (ChsEs), which use FAD as a coenzyme [64]. The 10 mol of FADH_2_ could be produced by converting one β-sitosterol molecule into AD. Thus, an additional 10 mol of H_2_O_2_ was produced during the complete degradation of the side chain of β-sitosterol [30]. The excessive accumulation of H_2_O_2_ has toxic effects on bacterial strains and hinders sterol metabolism. Microorganisms have two main antioxidant systems, including ROS scavenging enzymes, such as catalase (CAT), catalase-peroxidase, and superoxide dismutase, and low molecular weight (LMW) mercaptans, glutathione (GSH), fungal mercaptan (MSH), and ergothionine (EGT) [65,66]. The overexpression of ROS-scavenging enzymes or key enzymes for synthesizing LMW mercaptans is also the main technology for reducing intracellular ROS levels to obtain robust engineered strains [67,68].

### 3.5. Phytosterol-Side Chain Degradation and Energy Metabolism

Sterol-side chain degradation in *Mycolicibacterium* is catalyzed by multiple enzymes and produces a large number of high-energy compounds. There is an imbalance between energy supply and consumption in sterol conversion. Previous studies have shown that if the synthetic production is accompanied by ATP generation, the application of an ATP consumption strategy can improve strain production performance [69]. ATP consumption strategies include the direct action of F_O_F_1_-ATPase and the construction of the ATP futile cycle (AFC). The specific inhibition of F_O_F_1_-ATPase by the oligomycin and neomycin enhances the acetic acid, pyruvate and lactic acid yields of multiple microorganisms [70,71,72]. However, the use of inhibitors causes safety problems, and the genetic manipulation of F_O_F_1_-ATPase poses great risks [69]. The construction of AFC can effectively improve the yield of fermentation products. The AFC constructed in *E. coli* can enhance the secretion of pyruvate, acetic acid, lactate, and succinic acid [73,74,75]. Luo et al. (2019) [69] constructed an AFC in *Candida glabrata* reduced intracellular ATP levels by 51.0% and increased pyruvate production by 322.3%. Therefore, when product synthesis is accompanied by ATP formation, constructing an AFC to achieve forced ATP consumption and increase product synthesis is an effective metabolic transformation strategy.

## 4. Rational Construction of Recombinant *M. neoaurum* Based on NCMS

Metabolic engineering is an effective tool for improving the production capacity of strains and increasing productivity. Increasing metabolic fluxes and eliminating byproduct pathways by knocking out or introducing new key metabolic enzyme genes are commonly used transformation strategies in metabolic engineering. However, changes in intracellular metabolite levels and the microenvironment affect the metabolism of substances and energy [76]. Because of the complex metabolic network and regulatory mechanism in cells, the transformation of the core metabolic pathway cannot always achieve the expected effect. The modification and regulation of non-core metabolic pathways, such as substrate transport, intracellular redox balance, key intermediate metabolites, and energy load, will play a beneficial role.

### 4.1. Phytosterol Uptake Pathway Modification

The inefficient mass transfer caused by the low solubility of sterols is the main factor limiting the conversion efficiency. As shown in Table 1, enhancing sterol transport system and limiting the synthesis of cell wall and attachment layer are the main means to improve mass transfer efficiency at present. As the only energy provider of the Mce4 transporter, MceG is necessary for mycolicibacterial strains to utilize cholesterol and phytosterols. He et al. (2018) [32] introduced the MceG of *M. tuberculosis* into *Mycolicibacterium* sp. strain MS136-GAB (previously known as *Mycobacterium* sp. strain MS136) and coexpressed the YrbEA and YrbEB genes, which increased the yield of 9OH-AD by 20%. It was concluded that the Mce4 transporter plays an active role in the uptake and transport of sterols. Zhang et al. (2021) [77] coexpressed optimized *Vitreoscilla* hemoglobin (VHb) and MceG genes in *M*. sp. strain LZ2 (Msp) to alleviate dissolved oxygen and mass transfer constraints. Compared with Msp, Msp-vgb/mceG effectively improved growth, yield, and adaptability to dissolved oxygen. The *embC* is an important gene in cell wall synthesis. The deletion of *embC* resulted in a deficiency of lipoarabinomannan and a significant increase in cell permeability. After 72 h of conversion, the sterol substrate conversion efficiency was increased by about 52.4% [78]. The deletion of *fbpC3*, a key factor for the synthesis of m-AG and TDM, increased the 9-OHAD production by 21.3%. The combined deletion of *fbpC3* and *embC* further increased the 9-OHAD yield. After 96 h of bioconversion in industrial resting cells, the 9-OHAD yield of 11.2 g/L was achieved from phytosterols (20 g/L) [79]. Subsequently, the deletion of β-ketoacyl-acyl carrier protein synthase gene (*kasB*) in *M. neoaurum* ATCC 25795 led to a disorder in the mycolic acids ratio in the cell wall, and cell permeability increased by approximately two. The *kasB*-deletion strain increased 9-OHAD production by 137.7%. For resting cell conversion, the productivity of 9-OHAD reached 0.1135 g/L/h and the transformation time was shortened by 33% [80].

### 4.2. Metabolic Regulation Based on Coenzyme I

As the electron donor and acceptor of the substrate and the carrier of biohydrogen, the concentration of NADH/NAD^+^ determines the intracellular redox rate and balance. By regulating the concentration of NADH and NAD^+^, the physiological function and metabolic flow in cells can be changed in order to improve target product yield and productivity. Su et al. (2018) [81] increased the total amount of NAD^+^ and NADH by adding NAD^+^ as the precursor nicotinic acid and overexpressing nicotinic acid phosphoribosyltransferase (NAPRTase) in *M. neoaurum* TCCC 11978 (MNR M3); the AD (D) yield of MNR M3 was 9.6% higher than that of the parent strain. Further studies showed that the NAD^+^/NADH ratio first increased and then decreased with the prolongation of the conversion time during sterol to AD by MNR M3. NADH levels remained high, and the NAD^+^/NADH ratio was low at the later stages of transformation. At this time, the strain entered a low-metabolism state, which was not conducive to sterol transformation. The NAD^+^/NADH ratio was increased by enhancing NADH flavin reductase and NADH oxidase from *Lactobacillus brevis*. The intracellular NADH content of the modified strain M3N2 decreased significantly, the ratio of NAD^+^/NADH increased, and the conversion rate increased by 147% compared with that of the original strain [82]. However, it was found that the growth of the M3N2 strain was inhibited, which may have been due to the excessive consumption of NADH by NADH flavin reductase and NADH oxidase. Harbut et al. (2018) [83] showed that NDH-II plays a key role in maintaining the balance of NAD^+^/NADH in *M. tuberculosis* and that the inhibition of NDH-II can increase the ratio of NAD^+^/NADH. Zhou et al. (2019) [84] compared the proteome of MNR before and after sterol addition and found that the expression level of the NDH-1 subunit significantly decreased in the presence of phytosterol. However, the expression of NDH-II, with similar functions, was not affected. The NDH-II genes (*ndh*) from MNR and *M. fortuitum* (MFT) were successfully expressed in MNR (NdhN and NdhF), respectively. Compared with the MNR, the levels of NADH and NAD^+^ of NdhN and NdhF increased, and the ratio of NAD^+^/NADH increased from 3.93 to 5.91 and 10.96, respectively. Shao et al. (2019) [85] introduced NADH oxidase from *Bacillus subtilis* into *M. neoaurum* JC-12 to increase the availability of NAD^+^. The toxic effects of H_2_O_2_ on cell growth and sterol transformation were eliminated by the overexpression of catalase. Finally, the recombinant strain produced 9.66 g/L ADD in a 5 L fermenter and the yield increased by 80%. Wang et al. (2020) [86] replaced the nitrogen source in *M. neoaurum* R10 with ammonium nitrate. When the AD/HBC ratio increased from 2.1 to 5.5, the intracellular NADH/NAD^+^ ratio decreased by 59.5% and AD production increased accordingly.

### 4.3. Metabolic Regulation Based on Propionyl-CoA

As an acyl carrier group, coenzyme A activates the carbonyl groups of carboxylic acids in fatty acids and amino acids and forms a coenzyme A library composed of coenzyme A, succinyl-CoA, propionyl-CoA, and malonyl-CoA. There are more than 100 reactions related to coenzyme A in organisms, including the TCA cycle, fatty acid degradation, and the biosynthesis of metabolites of fatty acids, amino acids, and secondary metabolites [87]. The imbalance in coenzyme A homeostasis profoundly impacts cell metabolism. *M. neoaurum* produces acetyl-CoA and propionyl-CoA when phytosterol is used. The gene manipulation for mycolicibacterial strains aims to improve the efficiency of the side-chain degradation of sterols. Improving side chain degradation efficiency means that more short-chain molecules, such as propionyl-CoA and acetyl-CoA, are produced, which inevitably leads to the excessive accumulation of propionyl-CoA and produces toxic effects on cells, thus reducing productivity.

**Figure 3:**
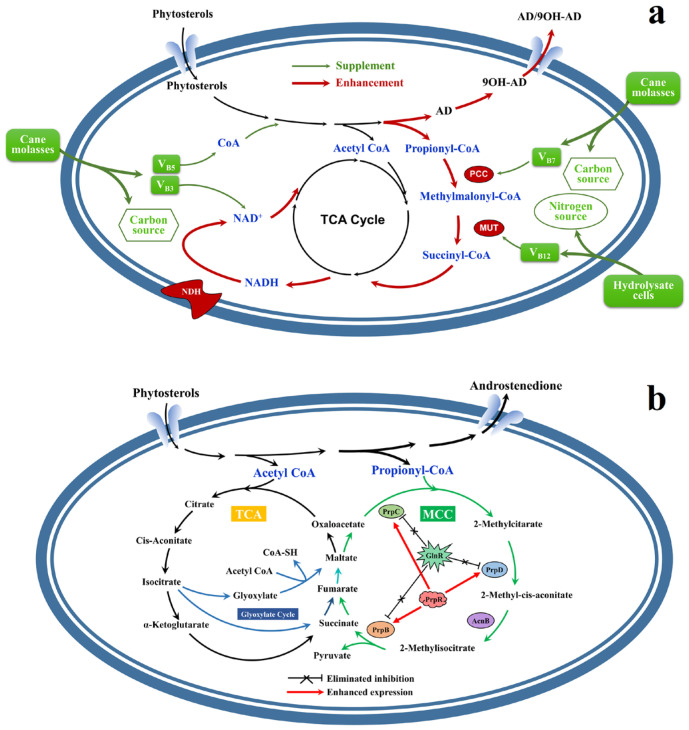
Construction scheme of androstenedione producing recombinant strain based on propionyl-CoA metabolism regulation. (**a**) Metabolic regulation strategy of propionyl coenzyme A based on MMC pathway; (**b**) Metabolic regulation strategy of propionyl coenzyme A based on MCC pathway.

The MMC pathway is essential for the metabolism of odd-chain fatty acids by *Mycolicibacterium* [88]. It has been reported that enhancing the MMC pathway in *Saccharopolyspora erythraea*, *Actinosynnema pretiosum*, and *Streptomyces hygroscopicus* can effectively increase the production of erythromycin, rapamycin, and ansamitocin [89]. Zhou et al. (2019b) [90] enhanced PCC in MNR and MFT (coexpressing NDH-II and PCCB recombinant strains: MNR-Fpcc-Fndh and MFT-Npcc-Nndh) and the production capacity of AD and 9-OHAD of recombinant strains was subsequently improved (Figure 3a). Compared to MNR, the ratio of NAD^+^/NADH in the recombinant strains was increased, and the conversion rate was effectively improved. Therefore, enhancing the MMC pathway and increasing the NAD^+^/NADH ratio could improve the AD yield of *M. neoaurum*.

In the MBPL pathway, methylmalonyl-CoA converted from propionyl-CoA was used in the synthesis of methyl-branched polyketide lipids [55]. Su et al. (2022) [41] enhanced the metabolism of propionyl-CoA to PDIM by overexpressing propionyl-CoA/acetyl-CoA carboxylase (AccA1) and polyketide synthase (AccD1) genes in the QC M3 (the deletion of 3-Ketosteroid-Δ1-dehydrogenase gene in the MNR) MBPL pathway. Transmission electron microscopy (TEM) analysis showed that the cell wall thickness of QC M3-A increased by 87%. The intracellular propionyl-CoA level decreased significantly after AraC overexpression. The tandem expression recombinant strain with inducible AccA1 expression and constitutive AraC expression was constructed. The strain with the highest AD conversion rate was 96.88% after 168 h of fermentation, which was 13.93% higher than that of the original strain [41].

The gene cluster of *prpB*, *prpC*, and *prpD* in the *Mycolicibacterium* genome is located in the same operon, known as the *prp* operon. The *prp* operon is necessary for *Mycolicibacterium* to grow on propionate as the only carbon source, and it plays a key role in the metabolism of propionyl-CoA [88]. Masiewicz et al. (2012) [91] identified a transcription factor (*prpR*) that regulates the MCC pathway in *M. tuberculosis* by inducing its own transcription and activating the *prp* operon. Liu et al. (2018) [92] found that GlnR can inhibit the transcription of the *prp* operon. GlnR regulates the MCC pathway by directly inhibiting *prp* operon transcription. It also demonstrated a correlation between nitrogen metabolism and the MCC pathway in *Mycolicibacterium*. Through genome mining and multisequence comparison, Zhang et al. (2020) [93] found that there is a complete MCC pathway in mycolicibacterial strains. The functions of the two regulatory factors were confirmed by overexpressing the *prp* operon gene and knocking out the *glnR* gene, and the corresponding metabolic regulation strategies were constructed (Figure 3b). The intracellular propionyl-CoA accumulation level of the modified MNR-prpR/ΔglnR strain decreased by 43% and cell viability increased by 22%. In addition, the adaptability of MNR-prpR/ΔglnR to low nitrogen sources was enhanced. At the 70% nitrogen source level, the highest AD conversion rate of MNR-prpR/glnR was 92.8%, 1.4 times that of the original strain.

### 4.4. Regulation Strategy of ROS Level

When mycolicibacterial strains use sterols, certain redox reactions occur in the cells, which require a large number of NAD^+^ and FAD molecules to produce ROS, exceeding the normal threshold [85]. NdhI is the main site of ROS production in prokaryotes, and the ROS produced by NDH-II is very weak compared to that of NdhI [94]. Zhou et al. (2019a) [84] measured the intracellular ROS levels and the cell viability of NDH-II-overexpressing and heterologously overexpressing strains (NdhN and NdhF). The results showed that the intracellular ROS levels of NdhN and NdhF were similar to those of MNR and increased significantly after 72 h; the accumulation of ROS was lower than that of MNR. At 144 h, ROS accumulation in MRN, NdhN, and NdhF was 7.747, 5.102, and 5.445 times that of MRN at 24 h, respectively. The activity analysis of the NdhN and NdhF strains showed that the activity of the NdhN and NdhF strains was always higher than that of MNR and reached the highest value at 96 h. Although the activity of the three strains decreased after 96 h, compared with MNR, NdhF still had higher activity. The enhancement of NDH-II in NdhF cells reduced ROS release by 42.32% and increased cell viability by 54.17%. Therefore, NDH-II is able to eliminate the excessive reduction in NADH and prevent ROS from being produced in large quantities to maintain the vitality of the strain. This is also the main reason for the enhancement of the NdhN and NdhF transformation abilities. In addition, the overexpression of *vgb* in Msp-vgb/mceG resulted in an intracellular ROS level of only 69% of that of Msp and had a smaller upward trend. Msp-vgb/mceG has a higher catalase activity than Msp, which may be the main reason why *vgb* is able to reduce ROS levels and improve strain activity [77].

To reduce the toxicity of ROS, Sun et al. (2019b) [64] conducted in-depth research on the ROS produced by *M. neoaurum* when catabolizing sterols. The detection of ROS in cells of the 9-OHAD-producing strain *M. neoaurum* NwIB-I and the 4-HBC-producing strain *M. neoaurum* WIII showed that the amount of ROS increased continuously during the fermentation process, accompanied by cell decline. ROS levels increased by 48.3% when phytosterol was used as the substrate. This indicates that the phytosterol transformation process is a process of excessive ROS production. There is a strong correlation between the increase in ROS levels and a decrease in cell viability, which is the limiting factor for phytosterol transformation. Seven ROS-induced genes, *per1*, *per2*, *trx*, *ahp*, *katG1*, *katG2*, and *cat* (encoding peroxiredoxin, thioredoxin reductase, alkyl hydroperoxide reductase, catalase-peroxidase, and catalase, respectively), were screened according to the transcriptome and proteome. Only CAT overexpression reduces intracellular ROS levels and promotes product synthesis. The ROS level of NwIB-I-*cat* increased by 21.3% after the addition of phytosterol. The 9-OHAD yield increased by 32.7% at 72 h and 12.6% at 120 h. The blocking of the LMW mercaptan (MSH and EGT) synthesis pathway in WIII showed that MSH and EGT were also essential for ROS control. To cope with other types of ROS, except H_2_O_2_, the synthesis pathway of LMW mercaptans (MSH and EGT), which are dominant in *Mycolicibacteria*, was strengthened. The overexpression of the key MSH genes *mshA*, *mshC*, and *mshD* decreased ROS levels and increased MSH production. Strain WIII-*mshA* showed the highest 4-HBC increase (17.2%) and decrease in ROS (26.8%). The overexpression of EGT synthetic gene clusters (*egtA*, *egtB*, *egtC*, *egtD*, and *egtE*) strain (WIII-*cluster*) can produce high levels of EGT. The coexpression of S-adenosylmethionine synthetase (SAM) and egtD also increased EGT production. The increase in EGT biosynthesis in the WiIII-*cluster* and WiIII-*egtD&SAM* decreased ROS levels by 23.3% and 28.9% and increased cell viability by 24.9% and 32.6%, respectively. Compared with WIII, the WIII-*cluster&SAM* (coexpressing the EGT synthetic gene cluster and SAM) ROS decreased by 30.8%, cell viability increased by 43.6%, and 4-HBC production increased by 32.1%. The recombinant strain WIII-*egt&msh&cat* was constructed by combining EGT synthetic gene clusters, *msh*, and *cat* expression. Compared to WIII, the intracellular ROS level of WIII-*egt&msh&cat* decreased by 46.9%, and cell viability increased by 54.2%. In sterol catabolism, the combination of CAT, MSH, and EGT was better able to control ROS and promote sterol conversion. Therefore, preventing and reducing intracellular ROS to improve cell viability is an effective strategy for improving sterol-transformed industrial strains’ yield and production efficiency.

### 4.5. Energy Metabolism Regulation Based on ATP

As an excellent producing strain, the transformation cycle of MNR and its modified strains is generally > 120 h. They also had a long AD production cycle, and the AD production rate showed a significant downward trend during the middle and late stages of production (after 72 h). AD production by *M. neoaurum* is a highly productive process. Zhou et al. (2020) [95] showed that after adding ATP and pyruvate to the fermentation process, the intracellular ATP level of MNR increased and greatly influenced AD production. Two AFCs were constructed at the pyruvate and oxaloacetate metabolic nodes. At the pyruvate node, pyruvate kinase (Pyk) catalyzes phosphoenolpyruvate to form pyruvate and produces an ATP molecule. Phosphoenolpyruvate synthase (Pps) converts pyruvate to phosphoenolpyruvate and ATP to AMP. At the oxaloacetate node, citrate synthase (CS) catalyzes the conversion of oxaloacetate and acetyl-CoA into citric acid and releases coenzyme A. The reverse reaction catalyzed by ATP-citrate lyase (Acl) consumes one ATP molecule to decompose citric acid into oxaloacetic acid. Therefore, one molecule of ATP is consumed for each cycle of conversion of citric acid and oxaloacetic acid in the empty cycle (CAFC) is composed of CS and Acl. Different intensities were introduced into AD-producing strains by constructing PpsA (MNR-P2 and MNR-P3) and Acl expression strains (MNR-C2 and MNR-C3). The AD conversion rates of MNR-P3 and MNR-C3 were 34% and 54% higher than those of MNR, respectively, and the highest AD-specific production rates (q_p_) were 1.2 and 1.7 times higher than those of MNR. The results showed that AFC significantly reduced ATP and propionyl-CoA levels, increased the NAD^+^/NADH ratio, and enhanced cell viability. qRT–PCR analysis showed that the key enzyme genes in the core metabolism and propionyl-CoA metabolism pathways were upregulated, and MNR-C3 was the most upregulated. The recombinant strains MNR-C3-pccB and MNR-C3-prpR were constructed by enhancing the MCC and MMC pathways of MNR-C3, respectively. Their highest conversion rates were 94.6% and 97.3%, respectively, and their maximum qp was 2.3 and 3.2 times that of the MNR (0.031). The enhancement of the propionyl-CoA metabolic pathway has a synergistic effect with AFC.

## 5. Enhancement of C-19 Steroid Yield by Improving the Medium Composition and Fermentation Biotechnology

### 5.1. Optimization of Transformation Medium

As a hydrophobic compound, phytosterol has a solubility in water less than 0.1 mmol/L [1]. Low water solubility limits the transformation of microorganisms, which is one of the main reasons for the long transformation time and low yield. The microbial conversion of steroids was found to be a process of mass transfer restrictions [96]. Although the micronization or derivatization of substrates can reduce the mass transfer limitation and improve the conversion yield, they are unsuitable for large-scale production owing to their complicated process and high cost. In addition, the cell walls and membranes of microorganisms are barriers for substrates to enter the cytoplasm, which also impacts the efficient operation of biotransformation. However, the reverse rate of free enzyme catalysis is 10–100 times faster than that of whole-cell catalysis [97]. Therefore, improving cell permeability is a method for enhancing transformation efficiency without affecting cell activity. Exogenous additives, such as vancomycin, glycine, and protamine, can change the structure of *Mycolicibacterium* cell walls and effectively improve the efficiency of steroid transformation [98]. However, their high cost of use has prompted researchers to focus on the screening of low-cost additives. The addition of additives such as surfactants, liquid polymers, lecithin, organic solvents, oils, ionic liquids, and cyclodextrins (CDs) can effectively improve the sterol conversion performance of strains [82,99]. Mancilla et al. (2018) [100] studied the effects of phytosterol oil-in-water (O/W) microdispersions with different particle sizes, culture media, and biotransformation conditions on AD yield. A phytosterol dispersion (20 g/L) with a particle size of 370 nm was transformed into *Mycolicibacterium* sp. B 3805 (previously known as *Mycobacterium* sp. B3805) for 11 d to yield 7.4 g/L AD. Zhou et al. (2019) [39] were the first to use polyoxyethylene (10) nonylphenyl ether (TX-40) for phytosterol biotransformation. Under 0.7% TX-40, the solubility of phytosterol increased by 12.1 times, and the 9-OHAD yield reached 42.5%, which was 217.2% higher than that of the control. The addition of TX-40 significantly downregulated the transcription levels of key lipid synthesis enzymes, changing the *M.* sp. LY-1 cell membrane composition and improving cell membrane permeability.

Introducing surfactants can improve steroid substrates’ solubility, but high concentrations of organic solvents often lead to the inhibition or deactivation of biocatalysts and environmental pollution. Using the special property of “hydrophilic outside the ring and hydrophobic inside the ring” of CDs, the inclusion of CDs with steroids can effectively improve the solubility of steroids in aqueous solution, thus improving the conversion efficiency of steroids by microorganisms. CDs are the most effective cosolvent for most producing strains to enhance the bioconversion of steroids, and the conversion rate of sterols can be increased 2–3 times by CDs [101,102]. Shtratnikova et al. (2017) [103] used the RNA-seq technique to study the transcription level changes of the steroid catabolic gene M. sp. VKM Ac-1817D in the presence of methyl-β-cyclodextrin (RM-β-CD). With phytosterol and MCD supplementation, most of the genes in KstR-regulon were slightly upregulated and those in KstR2-regulon decreased. Su et al. (2020) [104] used proteomic technology to explore whether hydroxypropyl-β-cyclodextrin (HP-β-CD) enhances the metabolism of phytosterols by *M. neoaurum* M3C2. The addition of HP-β-CD promoted the growth of M3C2 and increased the NAD^+^/NADH ratio. HP-β-CD alleviated the inhibitory effects of phytosterol metabolites on cell growth and the ETC pathway. The analysis of differentially expressed proteins showed that HP-β-CD significantly interfered with the expression of the M3C2 protein in the presence of phytosterols. Twenty-eight enzymes belonging to the core metabolic pathway of phytosterols were upregulated, thereby enhancing the biotransformation of phytosterols. However, most of the proteins involved in glucose metabolism and the TCA and ETC pathways related to energy metabolism were downregulated. This may be because CDs promote phytosterol degradation and produce acyl-CoA, which replaces glucose metabolism.

Although CDs have an excellent usage effect, their high cost makes their application in industrial production difficult. Soybean oil can improve steroid productivity by increasing matrix solubility and enhancing cell membrane permeability [105]. Su et al. (2017) [106] studied the mechanism by which soybean oil promotes AD production in MNRs. The results showed that when 16% (v/v) soybean oil was added as an oxygen carrier, the volumetric oxygen transfer coefficient value increased by 44%, and the AD yield increased by 84%. The analysis of intracellular cofactor levels showed that the levels of NAD^+^ and ATP were 27% and 43.75% higher than those of the control, respectively. Compared with the control the NADH levels and NADH/NAD^+^ ratios decreased by 72% and 79.5%, respectively. Adding soybean oil to the transformation system increased the solubility and conversion yield of phytosterols. As an oxygen carrier, it can also increase oxygen supply and regulate intracellular coenzyme I levels [106]. However, an addition as high as 15% increases the production cost; at the same time, it also increases the complexity of the downstream processes and the difficulty of product extraction. Waste cooking oil (WCO) is the main waste from cooking oil in food processing and can be obtained from large-scale fried food processing, restaurants, and home kitchens [107]. Many studies have shown that the conversion of WCO into biofuels and polyhydroxyalkanoates is economically and technically feasible [108]. Zhou et al. (2019b) [84] introduced 16 cosolvents into the transformation system of NdhF to evaluate the effects of single and complex solutes on phytosterol biotransformation. Compared to the control group, RM-β-CD and HP-β-CD had the best effects, followed by Tween 80 and Span 80. The effect of WCO is similar to that of soybean oil, but the cost of using WOC is half that of soybean oil and less than 1% that of RM-β-CD. Based on optimizing the addition of Tween 80 and WCO, a Tween 80 (0.4%)–WCO (14%)–water medium system (TOWS) was constructed. In the TOWS system, the conversion performances of MNR and NdhF were improved. By comparing the AD generation rate (q_p_) of MNR and NdhF, it can be seen that the highest qp of NdhF at 60 h (0.074) was 1.897 times that of MNR (0.039, 84 h). In addition, the highest q_p_ generation time of NdhF was 24 h shorter than that MNR. Therefore, the TOWS system is able to effectively improve production efficiency and reduce production costs, resulting in significant economic advantages.

### 5.2. Selection and Optimization of Carbon and Nitrogen Sources

Glucose and yeast powder were the main components of the medium, accounting for a relatively high share of the phytosterol biotransformation cost. Using inexpensive carbon and nitrogen sources or even organic waste to replace glucose and yeast powder has become the main method of reducing fermentation costs and realizing economic production.

Zhou et al. (2019) [90] confirmed that MNR-Fpcc-Fndh and MFT-Npcc-Nndh could effectively utilize hydrolyzed and untreated cane molasses. Additionally, *Mycolicibacterium* cell hydrolysate (HMC) is used as a nitrogen source for phytosterol biotransformation. MNR-Fpcc-Fndh and MTF-Npcc-Nndh used untreated cane molasses and HMC to biotransform phytosterols in TOWS (Figure 3a). The results showed that both MNR-Fpcc-Fndh and MFT-Fpcc-Fndh had the largest cell volume and highest conversion rate. Production cost analysis showed that the raw material cost of MNR-Fpcc-Fndh was reduced by 23.93%, whereas that of MFT-Npcc-Nndh was decreased by 41.95%. In MNR, the knockout of the nitrogen source regulator GlnR is able to promote the metabolic pathway of MCC and enhance the transformation of phytosterols. Zhang et al. (2020) [93] constructed *prpR* overexpression strains in MNRΔglnR. MNR-prpR/ΔglnR exhibited the highest production (Figure 3b). At low nitrogen levels, *glnR* deletion relieved the inhibition of *prpDBC* by *GlnR* and increased propionyl-CoA metabolism by MCC. When the nitrogen source was reduced by 30%, the AD conversion rate of MNR-prpR/ΔglnR was 28.4% higher than that of MNR, and the time required to produce the highest AD production rate was 24 h shorter than that of MNR.

Wang et al. (2019) [86] used nitrate instead of ammonium as a nitrogen source for *M. neoaurum* R10. Nitrate can act as both a nitrogen source and an electron acceptor in sterol biotransformation, which reduces the NADH/NAD^+^ ratio and leads to the transfer of metabolic flux to AD. Moreover, the AD-to-by-product HBC ratio increased from 2.1 to 5.5. Therefore, using nitrate as a nitrogen source is a simple method to increase AD production and reduce byproduct yield.

### 5.3. Construction and Improvement of Fermentation Technology

During the process of sterol transformation by *Mycolicibacterium*, the construction and improvement of fermentation technology are also important to ensure yield. A direct way to improve the production efficiency of fermentation products is to develop an appropriate fermentation technology to shorten fermentation time. Repeated batch fermentation can effectively shorten fermentation time and has been applied to produce many fermented products [109,110,111,112]. Repeated batch fermentation strategy has the advantages of only one seed culture, significantly shortening or eliminating the growth delay period. It has become an effective strategy to shorten the fermentation cycle and improve productivity [113]. Zhou et al. (2019b) [84] introduced repeated batch fermentation for steroid transformations. After 17 d, the phytosterol conversion rate of NdhF remained above 80%, and the production efficiency was 0.921 g/L/d, which was 13.75 times that of MNR. The maintenance of the cell viability of recombinant bacteria is key to the successful operation of repeated batch fermentation and the improvement of production intensity. MNR-C3-prpR uses untreated cane molasses as a carbon source to produce AD via repeated batch fermentation [95]. After six batches of operation for 20 days, the AD yield of the MNR-C3-prpR remained at 6.22 g/L. The average conversion rate of phytosterol to AD in the six batches was 94.2%. Repeated batch operations can improve the utilization rate of cane molasses and shorten the average batch production time from 144 h to 80 h. Tang et al. (2019) [114] established a step-wise biotransformation strategy, using recombinant *M. neoaurum* and *Pichia pastoris* to efficiently transform phytosterols into boldenone. In the side chain degradation stage of transforming phytosterols into ADD, semi-batch fermentation strategy is used, which shortens the total biotransformation process. A glucose supplementation strategy was adopted during the conversion of ADD into boldenone, which provided sufficient reducing power for the transformation. Under the optimized conditions of the two strategies, the yield of boldenone increased from 10% to 76%, and the total biotransformation cycle was shortened by 41.7%.

Cell immobilization technology has been widely used in the production of biological products as an effective means of overcoming the limitations of mass transfer, improving the stability of biocatalyst operation, protecting cells from toxic substrates or products, improving production efficiency, and reducing production costs [115]. Zhang et al. (2021) [77] immobilized Msp-vgb/mceG with bagasse as immobilized material. Under the same substrate level, immobilized cell fermentation resulted in higher biomass, yield and productivity than free cells. In addition, immobilized cell fermentation effectively improved the substrate tolerance of Msp-vgb/mceG. Therefore, it was immobilized and repeatedly fermented in batches. After 10 batches of repeated batch fermentation, Msp-vgb/mceG showed good production performance and cell viability. The fermentation time to reach 50% yield of each batch was reduced from 65 h in the first batch to 33 h in the fourth batch. Compared with single-batch immobilized cell fermentation, the total fermentation time was shortened from 60 days to 37 days by immobilized repeated batch fermentation and the average transformation efficiency was 0.069, which was 1.77 times that of free cells (0.039). Therefore, immobilized repeated batch fermentation is a potential fermentation process for high efficiency androsterone production.

Substrate catalysis using cultured nongrowing cells in a specific buffer or transformation system is called resting cell transformation. Compared to growing cell fermentation, resting cell transformation has the advantages of stable transformation conditions, strong specificity, high transformation efficiency, fewer byproducts, less contamination of miscellaneous bacteria, and the easy separation of products [1]. Xiong et al. (2020) [78] evaluated the 9-OHAD production capacity of the WIΔ*embC* strain with *embC* deleted using a resting cell transformation system. After 96 h of transformation, the yield of WIΔ*embC* increased from 8.9 g/L to 9.9 g/L, and the yield increased from 0.0927 g/L/h to 0.1031 g/L/h. While evaluating the resting cell transformation of the *kasB*-deleted strain WIΔkasB, the yield of 9-OHAD was 10.9 g/L, the molar yield was 69.5%, and the transformation time was shortened by 33% compared with the parent strain [80].

During the biotransformation of phytosterols, the degradation of the nucleus is one of the main problems to be solved in order to obtain a high yield. Xu et al. (2015) [116] studied the effect of temperature on the degradation of the nucleus. The culture temperature increased from 30 °C to 37 °C, and the degradation of the nucleus decreased from 39.9% to 17.6%. Based on this, a two-stage process regulation method for cell culture and resting cell transformation was adopted to transform AD [117]. At a substrate concentration of 50 g/L, the yield of Ad increased to 24.7 g/L, and the production efficiency was 6.18 g/L/d. These results indicated that the two-step method combining temperature control with resting cells has great potential for the industrial application of phytosterol biotransformation.

## 6. Comparison of the Influence of Various Cofactor Regulation Strategies on AD Production

In recent reviews, the background of AD-producing strains, types of substrates and treatment methods, yield, and other information have been outlined in detail [1,6]. In order to show more clearly the effects of different cofactor regulation strategies and transformation system production technology on AD production, the production yield and productivity of MNR and recombinant strains constructed based on different cofactor regulation strategies were compared (Table 2). Through this comparison, it was found that all cofactor regulation strategies were able to effectively improve the transformation yield of AD under the same transformation system. The effect of a single cofactor regulation strategy on AD transformation was as follows: NDH-II overexpression > PAFC > MCC enhancement > MMC enhancement. Among these, the coenzyme I regulation strategy based on NDH-II overexpression had the highest transformation yield in the TOWS system. The PAFC-based strategy for regulating energy metabolism also performed well, and the conversion yield obtained by it was 93.2%. Because PAFC consumes ATP and reduces intracellular NADH levels, it regulates the coenzyme I ratio. The combination of NDH-II overexpression and PAFC aggravates the imbalance in intracellular energy metabolism; therefore, it is not a desirable combination strategy. The enhancement of the propionyl-CoA metabolic pathway promotes the production of NADH, and the overexpression of NDH-II and the combination of AFC forms a cycle of NADH production and consumption, which provides a driving force for the degradation of the phytosterol-side chain. Therefore, constructing a multi-cofactor integrated regulation strategy combining NDH-II overexpression, PAFC, and propionyl-CoA metabolic pathway enhancement can further improve the transformation ability of the strain. The strategy of overexpression of NDH-II combined with the enhancement of MMC resulted in the highest conversion rate of AD of MNR-Fpcc-Fndh in the TOWS + molasses + HMC system (96.4%). The combination of PAFC and MCC enhancements resulted in the highest conversion rates of MNR-P3-prpR in the cyclodextrin and TOWS + molasses systems of 97.3% and 97%, respectively.

A comparison of productivity shows that applying a single cofactor control strategy or a comprehensive cofactor control strategy can greatly improve the productivity of AD. Applying the new transformation system TWOS and the repeated batch fermentation process resulted in several more improvements. The Ndh productivity in the TOWS system was 0.921 g/L/d by repeated batch fermentation. Repeated batch fermentation of MNR-C3-prpR with cane molasses as a carbon source in the TOWS system reached 1.960 g/L/d, which were 29.3, 4.5, and 4.7 times higher than those of MNR without additives, cyclodextrin, and TOWS systems, respectively. Therefore, the comprehensive control strategy of cofactors and the production using new conversion system constructed in this study is able to realize the efficient production of AD.

## 7. Concentrations and Future Considerations

Steroids are the second-largest class of drugs after antibiotics and play a prominent role in the pharmaceutical industry. C-19 steroids, especially AD, are the main raw materials for steroid hormone drugs. The global market for AD and ADD exceeds 1000 tons per year, and it is estimated that the global AD market will reach USD 210 million by 2025 [6]. Promoting the green, efficient, and low-cost production of C-19 steroids, such as AD, has become an important strategic demand for the global steroid pharmaceutical industry to achieve green, efficient production and sustainable development. The sterol core metabolic pathway of the producing strain was analyzed using high-throughput sequencing, proteomics, and other technical methods. Based on this, metabolic engineering transformation effectively improved the AD production performance of the strain. In recent years, significant progress has been made in NCMS research. It mainly focuses on the molecular mechanisms and genetic engineering of accelerating sterol transport uptake, regulating coenzyme I balance, strengthening propionyl-CoA metabolism, reducing ROS levels, and controlling energy metabolism. Some of the key enzymes of NCMS, such as NDH-II, PCC, GlnR, PrpR, AraC, NAT, CAT, EGT, and SAM, have been proven to be effective in enhancing sterol metabolism. In addition, optimizing the culture medium and constructing various fermentation processes further improved the productivity of the modified strain. Although many breakthroughs have been made in the research of NCMS, there are still many unsolved problems regarding the efficient conversion and production of C-19 steroids. Therefore, it is necessary to explore new research ideas and transformation schemes and, on the basis of analyzing the global metabolism and regulation mechanism of sterol metabolism by *Mycolicibacterium*, carry out precise metabolic regulation and construct the fine fermentation process regulation strategy to achieve the efficient production of strains.

Some existing NCMS research has not been combined with SCM metabolic transformation. If we combine these factors, we may play a synergistic role and further improve the production performance of the strain. Global transcriptional regulators such as GlnR and SigD have been shown to regulate sterol metabolism [93,118]. The regulatory mechanism of transcription factors related to phytosterol metabolism can be analyzed from the perspective of global metabolic regulation, locate the specific targets, and a global optimization strategy can be constructed, which can effectively improve the efficiency of steroid transformation by the strains. The classical pMV261 expression and p2NIL/pGOAL19 double plasmid knockout systems are used for most modifications of steroid production strains in intermediates. The resistance tag of pMV261 limits the application of recombinant strains for industrial production. Although the p2NIL/pGOAL19 double plasmid knockout system and the deletion and unlabeled insertion of specific genes have been realized, the operation process is cumbersome and inefficient, and an effective and efficient unlabeled insertion method of genes is urgently needed. We were delighted to find that Liu et al. (2022) [119] recently constructed a gene knockout system based on CRISPR–Cas12a, which can easily achieve targeted genome mutation without an exogenous donor template. Combined with the pNIL/pGOAL system, CRISPR–Cas12a can successfully integrate the genes of interest into targeted genomic sites. The CRISPR system shows great potential for accurate genome editing in *Mycolicibacterium* species. It provides an efficient and convenient research tool for exploring and transforming the regulatory mechanism of sterol metabolism in *Mycolicibacterium*.

Currently, most AD-derived drugs are synthesized by chemical methods, and much research is needed to realize the substitution of biological or enzymatic transformation. In addition to *Mycolicibacteria*, many species of microorganisms have been proven to have sterol degradation ability [120]. Different species have different sterol metabolic pathways. The pure culture of a single microorganism or metagenomic research on sterol-degrading microbial communities will expand the understanding of sterol metabolic pathways and enzymes. The microbial invertase gene sequence was obtained, cloned, and expressed, and the directional evolution of the enzyme was realized through enzyme engineering. A transformation system with high catalytic activity was constructed in a way that the biological method of chemical synthesis of AD-derived drugs is able to replace and expand the types of downstream drugs based on AD.

In addition, with the rapid development of synthetic biology, it is possible to synthesize new steroid intermediates and steroid drugs and even realize the de novo synthesis of steroid drugs. This can not only completely solve the problem of raw materials but also simplify the process route of steroid drug production and reduce the hazardous waste discharge and accident risk in the reaction process, thus greatly reducing the environmental protection pressure and production cost. Therefore, using synthetic biology to establish green and efficient microbial cell factories to produce steroid drugs (intermediates) is the mainstream direction for future technological system reform, which has extremely important practical significance for developing the steroid drug industry worldwide.

## 8. Conclusions

This review summarizes the research progress of molecular mechanisms and metabolic modifications related to NCMS. The results showed that accelerating sterol transport uptake, regulating coenzyme I balance, enhancing propionyl-CoA metabolism, reducing ROS levels, and regulating energy metabolism were all effective means to improve the production performance of the strain. In addition, the optimization of the transformation medium and culture medium and the application of biotechnology, such as repeated batch fermentation, immobilization, and resting cells, is able to expand the effect of NCMS regulation and improve the production efficiency of C-19 steroids.

## Figures and Tables

**Table 1:** Comparison of the effects of various promote sterol uptake strategies on phytosterols conversion.

Strains	Strategies to Promote Sterol Uptake	Substrate (g/L)	Cosolvent	Fermentation Biotechnology	Main Products	References
*Mycobacterium* sp. strain MS136	Enhancement of the Mce4 transport system by over-expressing *mceG*, yrbE4A and *yrbE4B*	13 g/L phytosterol	70 g/L β-cyclodextrin; 0.2 g/L Tween-80	Batch fermentation	6.0 g/L 9OH-AD	[32]
*Mycobacterium* sp. Strain LZ2	Coexpression of the optimized *Vitreoscilla* hemoglobin gene and *mceG*	10 g/L phytosterol	25 mM hydroxypropyl-β-cyclodextrin	Immobilized repeated batch fermentation	6.46 g/L AD	[77]
Deletion of kstD1, kstD2 and kstD3 in *M. neoaurum* ATCC 25795	Deleted the key gene *embC* required for the synthesis of lipoarabinomannan from lipomannan	20 g/L phytosterols	80 g/L hydrox-ypropyl-β-cyclodextrin	Batch fermentation of resting cell	9.9 g/L 9-OHAD	[78]
Deletion of kstD1, kstD2 and kstD3 in *M. neoaurum* ATCC 25795	Deletion *fbpC3* (a key factor for the synthesis of m-AG and TDM) and *embC*	20 g/L phytosterols	80 g/L hydrox-ypropyl-β-cyclodextrin	Batch fermentation of resting cell	11.2 g/L 9-OHAD	[79]
Deletion of kstD1, kstD2 and kstD3 in *M. neoaurum* ATCC 25795	Deletion *kasB* (β-ketoacyl-acyl carrier protein synthase gene)	20 g/L phytosterols	80 g/L hydrox-ypropyl-β-cyclodextrin	Batch fermentation of resting cell	10.9 g/L 9-OHAD	[80]

**Table 2:** Comparison of the effects of various cofactor regulation strategies on AD production.

Cofactor Regulation Strategies	Strain	Translational Systems	Conversion Rate (%)	Productivity (g/L/d)
NDH-II Overexpress	NdhF	None	23.9	0.166
NDH-II Overexpress	NdhF	TOWS	95.0	0.656
NDH-II Overexpress	NdhF	TOWS + Repeated batch	86.1	0.921
MMC Enhance	MNR-Fpcc	None	25.4	0.176
NDH-II Overexpress + MMC Enhance	MNR-Fpcc-Fndh	None	33.8	0.235
NDH-II Overexpress + MMC Enhance	MNR-Fpcc-Fndh	TOWS + Cane molasses + HMC	96.4	0.669
MCC Enhance	MNR-prpR	HP-β-CD	90.6	0.628
GlnR Knockout + MCC Enhance	MNR-prpDBC/ΔglnR	HP-β-CD	94.3	0.654
GlnR Knockout + MCC Enhance	MNR-prpDBC/ΔglnR	Low nitrogen sources + HP-β-CD	92.8	0.644
CAFC	MNR-P3	HP-β-CD	81.4	0.942
PAFC	MNR-C3	HP-β-CD	93.2	1.078
PAFC + MMC Enhance	MNR-P3-pccB	HP-β-CD	94.6	1.187
PAFC + MCC Enhance	MNR-P3-prpR	HP-β-CD	97.3	1.222
PAFC + MCC Enhance	MNR-P3-prpR	TOWS + Cane molasses	97.0	1.218
PAFC + MCC Enhance	MNR-P3-prpR	TOWS + Cane molasses + Repeated batch	94.2	1.96
None	MNR	None	11.5	0.067
None	MNR	HP-β-CD	75.7	0.439
None	MNR	TOWS	72.4	0.417

## Data Availability

Not applicable.

## References

[1] Zhao A., Zhang X., Li Y., Wang Z., Lv Y., Liu J., Alam M.A., Xiong W., Xu J. (2021). Mycolicibacterium cell factory for the production of steroid-based drug intermediates. Biotechnol. Adv..

[2] Seth D., Kamat D. (2019). Intranasal steroid therapy for allergic rhinitis. Pediatr. Ann..

[3] Fernandez-Cabezon L., Galán B., García J.L. (2018). New insights on steroid biotechnology. Front. Microbiol..

[4] Malaviya A., Gomes J. (2008). Androstenedione production by biotransformation of phytosterols. Bioresour. Technol..

[5] Xie R., Shen Y., Qin N., Wang Y., Su L., Wang M. (2015). Genetic differences in ksdD influence on the ADD/AD ratio of Myco-bacterium neoaurum. J. Ind. Microbiol. Biotechnol..

[6] Nunes V.O., Vanzellotti N.D.C., Fraga J.L., Pessoa F.L.P., Ferreira T.F., Amaral P.F.F. (2022). Biotransformation of phytosterols into androstenedione—A technological prospecting study. Molecules.

[7] Rodríguez-García A., Fernández-Alegre E., Morales A., Sola-Landa A., Lorraine J., Macdonald S., Dovbnya D., Smith M.C.M., Donova M., Barreiro C. (2016). Complete genome sequence of ‘Mycobacterium neoaurum’ NRRL B-3805, an androstenedione (AD) producer for industrial biotransformation of sterols. J. Biotechnol..

[8] Peng H., Wang Y., Jiang K., Chen X., Zhang W., Zhang Y., Deng Z., Qu X. (2020). A dual role reductase from phytosterols ca-tabolism enables the efficient production of valuable steroid precursors. Angew. Chemie. Int. Ed..

[9] Bragin E.Y., Shtratnikova V.Y., Dovbnya D.V., Schelkunov M.I., Pekov Y.A., Malakho S.G., Egorova O.V., Ivashina T.V., Sokolov S.L., Ashapkin V.V. (2013). Comparative analysis of genes encoding key steroid core oxidation enzymes in fast-growing *Mycobacterium* spp. Strains. J. Steroid Biochem. Mol. Biol..

[10] Zhang R., Liu X., Wang Y., Han Y., Sun J., Shi J., Zhang B. (2018). Identification, function, and application of 3-ketosteroid Δ1-dehydrogenase isozymes in *Mycobacterium neoaurum* DSM 1381 for the production of steroidic synthons. Microb. Cell Fact..

[11] Yao K., Xu L.Q., Wang F.Q., Wei D.Z. (2014). Characterization and engineering of 3-ketosteroid-△1-dehydrogenase and 3-ketosteroid-9α-hydroxylase in *Mycobacterium neoaurum* ATCC 25795 to produce 9α-hydroxy-4-androstene-3,17-dione through the catabolism of sterols. Metab. Eng..

[12] Shao M., Zhang X., Rao Z., Xu M., Yang T., Li H., Xu Z., Yang S. (2016). A mutant form of 3-ketosteroid-Δ1-dehydrogenase gives altered androst-1,4-diene-3, 17-dione/androst-4-ene-3,17-dione molar ratios in steroid biotransformations by *Mycobacterium neoaurum* ST-095. J. Ind. Microbiol. Biotechnol..

[13] Xiong L.-B., Liu H.-H., Xu L.-Q., Sun W.-J., Wang F.-Q., Wei D.-Z. (2017). Improving the production of 22-hydroxy-23,24-bisnorchol-4-ene-3-one from sterols in *Mycobacterium neoaurum* by increasing cell permeability and modifying multiple genes. Microb. Cell Factories.

[14] Lobastova T.G., Khomutov S.M., Shutov A.A., Donova M.V. (2019). Microbiological synthesis of stereoisomeric 7(α/β)-hydroxytestololactones and 7(α/β)-hydroxytestolactones. Appl. Microbiol. Biotechnol..

[15] Wang X., Feng J., Zhang D., Wu Q., Zhu D., Ma Y. (2017). Characterization of new recombinant 3-ketosteroid-Δ1-dehydrogenases for the biotransformation of steroids. Appl. Microbiol. Biotechnol..

[16] Chang H., Zhang H., Zhu L., Zhang W., You S., Qi W., Qian J., Su R., He Z. (2020). A combined strategy of metabolic pathway regulation and two-step bioprocess for improved 4-androstene-3,17-dione production with an engineered *Mycobacterium neoaurum*. Biochem. Eng. J..

[17] Gupta R.S., Lo B., Son J. (2018). Phylogenomics and comparative genomic studies robustly support division of the genus mycobacterium into an emended genus mycobacterium and four novel genera. Front. Microbiol..

[18] Oren A., Garrity G.M. (2016). List of new names and new combinations previously effectively, but not validly, published. Int. J. Syst. Evol. Microbiol..

[19] Malaviya A., Gomes J. (2009). Rapid screening and isolation of a fungus for sitosterol to androstenedione biotransformation. Appl. Biochem. Biotechnol..

[20] Lin Y., Song X., Fu J., Lin J., Qu Y. (2009). Microbial transformation of phytosterol in corn flour and soybean flour to 4-androstene-3,17-dione by *Fusarium moniliforme* sheld. Bioresour. Technol..

[21] Liu Y., Chen G., Ge F., Li W., Zeng L., Cao W. (2011). Efficient biotransformation of cholesterol to androsta-1,4-diene-3,17-dione by a newly isolated actinomycete *Gordonia neofelifaecis*. World J. Microbiol. Biotechnol..

[22] Chaudhari P.N., Chaudhari B.L., Chincholkar S.B. (2010). Cholesterol biotransformation to androsta-1,4-diene-3,17-dione by growing cells of *Chryseobacterium gleum*. Biotechnol. Lett..

[23] Donova M.V., Egorova O. (2012). Microbial steroid transformations: Current state and prospects. Appl. Microbiol. Biotechnol..

[24] Yao K., Wang F.-Q., Zhang H.-C., Wei D.-Z. (2013). Identification and engineering of cholesterol oxidases involved in the initial step of sterols catabolism in *Mycobacterium neoaurum*. Metab. Eng..

[25] Uhía I., Galán B., Morales V., García J.L. (2011). Initial step in the catabolism of cholesterol by *Mycobacterium smegmatis* mc2155. Environ. Microbiol..

[26] Horinouchi M., Kurita T., Hayashi T., Kudo T. (2010). Steroid degradation genes in *Comamonas testosteroni* TA441: Isolation of genes encoding a Δ4(5)-isomerase and 3α- and 3β-dehydrogenases and evidence for a 100kb steroid degradation gene hot spot. J. Steroid Biochem. Mol. Biol..

[27] Ivashina T.V., Nikolayeva V.M., Dovbnya D.V., Donova M.V. (2012). Cholesterol oxidase ChoD is not a critical enzyme accounting for oxidation of sterols to 3-keto-4-ene steroids in fast-growing *Mycobacterium* sp. VKM Ac-1815D. J. Steroid Biochem. Mol. Biol..

[28] Kreit J. (2017). Microbial catabolism of sterols: Focus on the enzymes that transform the sterol 3 beta-hydroxy-5-en into 3-keto-4-en. FEMS Microbiol. Lett..

[29] Lu R., Schaefer C.M., Nesbitt N.M., Kuper J., Kisker C., Sampson N.S. (2017). Catabolism of the cholesterol side chain in *Mycobacterium tuberculosis* is controlled by a redox-sensitive thiol switch. ACS Infect. Dis..

[30] Szentirmai A. (1990). Microbial physiology of sidechain degradation of sterols. J. Ind. Microbiol. Biotechnol..

[31] Xu L.-Q., Liu Y.-J., Yao K., Liu H.-H., Tao X.-Y., Wang F.-Q., Wei D.-Z. (2016). Unraveling and engineering the production of 23,24-bisnorcholenic steroids in sterol metabolism. Sci. Rep..

[32] He K., Sun H., Song H. (2018). Engineering phytosterol transport system in *Mycobacterium* sp. strain MS136 enhances production of 9α-hydroxy-4-androstene-3,17-dione. Biotechnol. Lett..

[33] Rank L., Herring L.E., Braunstein M. (2021). Evidence for the mycobacterial Mce4 transporter being a multiprotein complex. J. Bacteriol..

[34] Perkowski E.F., Miller B.K., McCann J.R., Sullivan J.T., Malik S., Allen I.C., Godfrey V., Hayden J.D., Braunstein M. (2016). An orphaned Mce-associated membrane protein of *Mycobacterium tuberculosis* is a virulence factor that stabilizes Mce transporters. Mol. Microbiol..

[35] Nazarova E.V., Montague C.R., La T., Wilburn K.M., Sukumar N., Lee W., Caldwell S., Russell D.G., VanderVen B.C. (2017). Rv3723/LucA coordinates fatty acid and cholesterol uptake in *Mycobacterium tuberculosis*. Elife.

[36] Pandey A.K., Sassetti C.M. (2008). Mycobacterial persistence requires the utilization of host cholesterol. Proc. Natl. Acad. Sci. USA.

[37] Tahlan K., Wilson R., Kastrinsky D.B., Arora K., Nair V., Fischer E., Barnes S.W., Walker J.R., Alland D., Barry C.E. (2012). SQ109 targets MmpL3, a membrane transporter of trehalose monomycolate involved in mycolic acid donation to the cell wall core of mycobacterium tuberculosis. Antimicrob. Agents Chemother..

[38] Abuhammad A. (2017). Cholesterol metabolism: A potential therapeutic target in *Mycobacteria*. Br. J. Pharmacol..

[39] Zhou L., Li H., Xu Y., Liu W., Zhang X., Gong J., Xu Z., Shi J. (2019). Effects of a nonionic surfactant TX-40 on 9α-hydroxyandrost-4-ene-3,17-dione biosynthesis and physiological properties of *Mycobacterium* sp. LY-1. Process. Biochem..

[40] Queiroz A., Medina-Cleghorn D., Marjanovic O., Nomura D.K., Riley L.W. (2015). Comparative metabolic profiling of mce1 operon mutant vs. wild-type *Mycobacterium tuberculosis* strains. Pathog. Dis..

[41] Su Z., Zhang Z., Yu J., Yuan C., Shen Y., Wang J., Su L., Wang M. (2022). Combined enhancement of the propionyl-CoA metabolic pathway for efficient androstenedione production in *Mycolicibacterium neoaurum*. Microb. Cell Fact..

[42] Sedlaczek L., Lisowska K., Korycka M., Rumijowska A., Ziółkowski A., Długoński J. (1999). The effect of cell wall components on glycine-enhanced sterol side chain degradation to androstene derivatives by mycobacteria. Appl. Microbiol. Bio-Technol..

[43] Korkegian A., Roberts D.M., Blair R., Parish T. (2014). Mutations in the essential arabinosyltransferase EmbC lead to alterations in *Mycobacterium tuberculosis* lipoarabinomannan. J. Biol. Chem..

[44] Anderson D.H., Harth G., Horwitz M.A., Eisenberg D. (2001). An interfacial mechanism and a class of inhibitors inferred from two crystal structures of the Mycobacterium tuberculosis 30 kda major secretory protein (antigen 85B), a mycolyl transferase. J. Mol. Biol..

[45] Goins C.M., Dajnowicz S., Smith M.D., Parks J.M., Ronning D.R. (2018). Mycolyltransferase from *Mycobacterium tuberculosis* in covalent complex with tetrahydrolipstatin provides insights into antigen 85 catalysis. J. Biol. Chem..

[46] Warrier T., Tropis M., Werngren J., Diehl A., Gengenbacher M., Schlegel B., Schade M., Oschkinat H., Daffe M., Hoffner S. (2012). Antigen 85C inhibition restricts mycobacterium tuberculosis growth through disruption of cord factor biosynthesis. Antimicrob. Agents Chemother..

[47] Yang X., Nesbitt N.M., Dubnau E., Smith I., Sampson N.S. (2009). Cholesterol metabolism increases the metabolic pool of propionate in *Mycobacterium tuberculosis*. Biochemistry.

[48] Griffin J.E., Pandey A.K., Gilmore S.A., Mizrahi V., Mckinney J.D., Bertozzi C.R., Sassetti C.M. (2012). Cholesterol catabolism by *Mycobacterium tuberculosis* requires transcriptional and metabolic adaptations. Chem. Biol..

[49] Gopinath K., Moosa A., Mizrahi V., Warner D.F. (2013). Vitamin B 12 metabolism in Mycobacterium tuberculosis. Future Microbiol..

[50] Resh M. (2013). Lipid Modification of Proteins: Targeting to Membranes.

[51] Ouellet H., Johnston J.B., de Montellano P.R.O. (2011). Cholesterol catabolism as a therapeutic target in *Mycobacterium tuberculosis*. Trends Microbiol..

[52] Puckett S., Trujillo C., Wang Z., Eoh H., Ioerger T.R., Krieger I., Sacchettini J., Schnappinger D., Rhee K.Y., Ehrt S. (2017). Glyox-ylate detoxification is an essential function of malate synthase required for carbon assimilation in *Mycobacterium tuberculosis*. Proc. Natl. Acad. Sci. USA.

[53] Lyonnet B.B., Diacovich L., Cabruja M., Bardou F., Quémard A., Gago G., Gramajo H. (2014). Pleiotropic effect of AccD5 and AccE5 depletion in acyl-coenzyme A carboxylase activity and in lipid biosynthesis in mycobacteria. PLoS ONE.

[54] Eoh H., Rhee K.Y. (2014). Methylcitrate cycle defines the bactericidal essentiality of isocitrate lyase for survival of *Mycobacterium tuberculosis* on fatty acids. Proc. Natl. Acad. Sci. USA.

[55] Singh P., Sinha R., Tyagi G., Sharma N.K., Saini N.K., Chandolia A., Prasad A.K., Varma-Basil M., Bose M. (2018). PDIM and SL1 accumulation in *Mycobacterium tuberculosis* is associated with mce4A expression. Gene.

[56] Claes W.A., Puhler A., Kalinowski J. (2002). Identification of two *prpDBC* gene clusters in *Corynebacterium glutamicum* and their involvement in propionate degradation via the 2-methylcitrate cycle. J. Bacteriol..

[57] Textor S., Wendisch V.F., de Graaf A., Müller U., Linder M.I., Linder D., Buckel W. (1997). Propionate oxidation in *Escherichia coli*: Evidence for operation of a methylcitrate cycle in bacteria. Arch. Microbiol..

[58] Borisov V., Siletsky S., Nastasi M., Forte E. (2021). ROS defense systems and terminal oxidases in bacteria. Antioxidants.

[59] Mendes-Ferreira A., Sampaio-Marques B., Barbosa C., Rodrigues F., Costa V., Mendes-Faia A., Ludovico P., Leao C. (2010). Ac-cumulation of non-superoxide anion reactive oxygen species mediates nitrogen-limited alcoholic fermentation by *Saccharomyces cerevisiae*. Appl. Environ. Microbiol..

[60] Kerscher S., Dröse S., Zickermann V., Brandt U. (2008). The three families of respiratory NADH dehydrogenases. Results Probl. Cell Differ..

[61] Muller F.L., Liu Y., Abdul-Ghani M.A., Lustgarten M.S., Bhattacharya A., Jang Y.C., Van Remmen H. (2008). High rates of superoxide production in skeletal-muscle mitochondria respiring on both complex I- and complex II-linked substrates. Biochem. J..

[62] Kussmaul L., Hirst J. (2006). The mechanism of superoxide production by NADH: Ubiquinone oxidoreductase (complex I) from bovine heart mitochondria. Proc. Natl. Acad. Sci. USA.

[63] Knuuti J., Belevich G., Sharma V., Bloch D.A., Verkhovskaya M. (2013). A single amino acid residue controls ROS production in the respiratory complex I from *Escherichia coli*. Mol. Microbiol..

[64] Sun W.-J., Wang L., Liu H.-H., Liu Y.-J., Ren Y.-H., Wang F.-Q., Wei D.-Z. (2019). Characterization and engineering control of the effects of reactive oxygen species on the conversion of sterols to steroid synthons in *Mycobacterium neoaurum*. Metab. Eng..

[65] Richard-Greenblatt M., Bach H., Adamson J., Peña-Diaz S., Li W., Steyn A.J., Av-Gay Y. (2015). Regulation of ergothioneine biosynthesis and its effect on *Mycobacterium tuberculosis* growth and infectivity. J. Biol. Chem..

[66] Servillo L., Castaldo D., Casale R., D’Onofrio N., Giovane A., Cautela D., Balestrieri M.L. (2015). An uncommon redox behavior sheds light on the cellular antioxidant properties of ergothioneine. Free. Radic. Biol. Med..

[67] Si M., Zhao C., Zhang B., Wei D., Chen K., Yang X., Xiao H., Shen X. (2016). Overexpression of mycothiol disulfide reductase enhances *Corynebacterium glutamicum* robustness by modulating cellular redox homeostasis and antioxidant proteins under oxidative stress. Sci. Rep..

[68] Nakajima S., Satoh Y., Yanashima K., Matsui T., Dairi T. (2015). Ergothioneine protects *Streptomyces coelicolor* A3(2) from oxidative stresses. J. Biosci. Bioeng..

[69] Luo Z., Zeng W., Du G., Chen J., Zhou J. (2019). Enhanced pyruvate production in *Candida glabrata* by engineering ATP futile cycle system. ACS Synth. Biol..

[70] Johnson K.M., Cleary J., Fierke C.A., Opipari A.W., Glick G.D. (2006). Mechanistic basis for therapeutic targeting of the mito-chondrial F 1 F o-ATPase. ACS Chem. Biol..

[71] Boecker S., Zahoor A., Schramm T., Link H., Klamt S. (2019). Broadening the scope of enforced ATP wasting as a tool for metabolic engineering in *Escherichia coli*. Biotechnol. J..

[72] Liu J., Kandasamy V., Würtz A., Jensen P.R., Solem C. (2016). Stimulation of acetoin production in metabolically engineered *Lactococcus lactis* by increasing ATP demand. Appl. Microbiol. Biotechnol..

[73] Chao Y.P., Liao J.C. (1994). Metabolic responses to substrate futile cycling in *Escherichia coli*. J. Biol. Chem..

[74] Hädicke O., Bettenbrock K., Klamt S. (2015). Enforced ATP futile cycling increases specific productivity and yield of anaerobic lactate production in *Escherichia coli*. Biotechnol. Bioeng..

[75] Li J., Li Y., Cui Z., Liang Q., Qi Q. (2017). Enhancement of succinate yield by manipulating NADH/NAD+ ratio and ATP generation. Appl. Microbiol. Biotechnol..

[76] Chubukov V., Gerosa L., Kochanowski K., Sauer U. (2014). Coordination of microbial metabolism. Nat. Rev. Genet..

[77] Zhang Y., Zhou X., Yao Y., Xu Q., Shi H., Wang K., Feng W., Shen Y. (2021). Coexpression of VHb and MceG genes in *Mycobacterium* sp. strain LZ2 enhances androstenone production via immobilized repeated batch fermentation. Bioresour. Technol..

[78] Xiong L.-B., Liu H.-H., Song X.-W., Meng X.-G., Liu X.-Z., Ji Y.-Q., Wang F.-Q., Wei D.-Z. (2020). Improving the biotransformation of phytosterols to 9α-hydroxy-4-androstene-3,17-dione by deleting embC associated with the assembly of cell envelope in *Mycobacterium neoaurum*. J. Biotechnol..

[79] Xiong L.-B., Liu H.-H., Song L., Dong M.-M., Ke J., Liu Y.-J., Liu K., Zhao M., Wang F.-Q., Wei D.-Z. (2022). Improving the bio-transformation efficiency of soybean phytosterols in *Mycolicibacterium neoaurum* by the combined deletion of fbpC3 and embC in cell envelope synthesis. Synth. Syst. Biotechnol..

[80] Xiong L.-B., Liu H.-H., Zhao M., Liu Y.-J., Song L., Xie Z.-Y., Xu Y.-X., Wang F.-Q., Wei D.-Z. (2020). Enhancing the bioconversion of phytosterols to steroidal intermediates by the deficiency of kasB in the cell wall synthesis of *Mycobacterium neoaurum*. Microb. Cell Factories.

[81] Su L., Shen Y., Gao T., Cui L., Luo J., Wang M. (2018). Regulation of NAD (H) pool by overexpression of nicotinic acid phosphoribosyltransferase for AD (D) production in *Mycobacterium neoaurum*. Lect. Notes Electr. Eng..

[82] Su L., Shen Y., Zhang W., Gao T., Shang Z., Wang M. (2017). Cofactor engineering to regulate NAD+/NADH ratio with its ap-plication to phytosterols biotransformation. Microb. Cell Fact..

[83] Harbut M.B., Yang B., Liu R., Yano T., Vilchèze C., Cheng B., Lockner J., Guo H., Yu C., Franzblau S.G. (2018). Small molecules targeting mycobacterium tuberculosis type II NADH dehydrogenase exhibit antimycobacterial activity. Angew. Chemie. Int. Ed..

[84] Zhou X., Zhang Y., Shen Y., Zhang X., Xu S., Shang Z., Xia M., Wang M. (2019). Efficient production of androstenedione by re-peated batch fermentation in waste cooking oil media through regulating NAD+/NADH ratio and strengthening cell vitality of *Mycobacterium neoaurum*. Bioresour. Technol..

[85] Shao M., Zhao Y., Liu Y., Yang T., Xu M., Zhang X., Rao Z. (2019). Intracellular environment improvement of *Mycobacterium neoaurum* for enhancing androst-1,4-diene-3,17-dione production by manipulating NADH and reactive oxygen species levels. Molecules.

[86] Wang X., Chen R., Wu Y., Wang D., Wei D. (2020). Nitrate metabolism decreases the steroidal alcohol byproduct compared with ammonium in biotransformation of phytosterol to androstenedione by *Mycobacterium neoaurum*. Appl. Biochem. Biotechnol..

[87] Leonardi R., Zhang Y.-M., Rock C.O., Jackowski S. (2005). Coenzyme A: Back in action. Prog. Lipid Res..

[88] Upton A.M., McKinney J.D. (2007). Role of the methylcitrate cycle in propionate metabolism and detoxification in *Mycobacterium smegmatis*. Microbiology.

[89] Zhao M., Fan Y., Wei L., Hu F., Hua Q. (2017). Effects of the methylmalonyl-CoA metabolic pathway on ansamitocin production in *Actinosynnema pretiosum*. Appl. Biochem. Biotechnol..

[90] Zhou X., Zhang Y., Shen Y., Zhang X., Zhang Z., Xu S., Luo J., Xia M., Wang M. (2019). Economical production of androstenedi-one and 9A-hydroxyandrostenedione using untreated cane molasses by recombinant mycobacteria. Bioresour. Technol..

[91] Masiewicz P., Brzostek A., Wolański M., Dziadek J., Zakrzewska-Czerwińska J. (2012). A novel role of the PrpR as a transcription factor involved in the regulation of methylcitrate pathway in *Mycobacterium tuberculosis*. PLoS ONE.

[92] Liu X.-X., Shen M.-J., Liu W.-B., Ye B.-C. (2018). GlnR-mediated regulation of short-chain fatty acid assimilation in *Mycobacterium smegmatis*. Front. Microbiol..

[93] Zhang Y., Zhou X., Wang X., Wang L., Xia M., Luo J., Shen Y., Wang M. (2020). Improving phytosterol biotransformation at low nitrogen levels by enhancing the methylcitrate cycle with transcriptional regulators PrpR and GlnR of *Mycobacterium neoaurum*. Microb. Cell Factories.

[94] Sena F.V., Sousa F.M., Oliveira A.S.F., Soares C.M., Catarino T., Pereira M.M. (2018). Regulation of the mechanism of type-II NADH: Quinone oxidoreductase from *S. aureus*. Redox Biol..

[95] Zhou X., Zhang Y., Shen Y., Zhang X., Zan Z., Xia M., Luo J., Wang M. (2020). Efficient repeated batch production of androstenedione using untreated cane molasses by *Mycobacterium neoaurum* driven by ATP futile cycle. Bioresour. Technol..

[96] Chen K.-C., Wey H.-C. (1990). Dissolution-enzyme kinetics of 11β-hydroxylation of cortexolone by *Curvularia lunata*. Enzym. Microb. Technol..

[97] Cook G.M., Hards K., Vilchèze C., Hartman T., Berney M. (2014). Energetics of respiration and oxidative phosphorylation in mycobacteria. Microbiol. Spectr..

[98] Sun W.-J., Liu Y.-J., Liu H., Ma J., Ren Y., Wang F., Wei D. (2019). Enhanced conversion of sterols to steroid synthons by augmenting the peptidoglycan synthesis gene pbpB in *Mycobacterium neoaurum*. J. Basic Microbiol..

[99] Li H., Fu Z., Li H., Zhang X., Shi J., Xu Z. (2014). Enhanced biotransformation of dehydroepiandrosterone to 3β,7α,15α-trihydroxy-5-androsten-17-one with Gibberella intermedia CA3-1 by natural oils addition. J. Ind. Microbiol. Biotechnol..

[100] Mancilla R.A., Little C., Amoroso A. (2017). Efficient bioconversion of high concentration phytosterol microdispersion to 4-Androstene-3,17-dione (AD) by *Mycobacterium* sp. B3805. Appl. Biochem. Biotechnol..

[101] Shen Y., Liang J., Li H., Wang M. (2014). Hydroxypropyl-β-cyclodextrin-mediated alterations in cell permeability, lipid and protein profiles of steroid-transforming Arthrobacter simplex. Appl. Microbiol. Biotechnol..

[102] Shen Y., Wang M., Zhang L., Ma Y., Ma B., Zheng Y., Liu H., Luo J. (2011). Effects of hydroxypropyl-β-cyclodextrin on cell growth, activity, and integrity of steroid-transforming Arthrobacter simplex and *Mycobacterium* sp. Appl. Microbiol. Biotechnol..

[103] Shtratnikova V.Y., Schelkunov M.I., Dovbnya D.V., Bragin E.Y., Donova M.V. (2017). Effect of methyl-β-cyclodextrin on gene expression in microbial conversion of phytosterol. Appl. Microbiol. Biotechnol..

[104] Su L., Xu S., Shen Y., Xia M., Ren X., Wang L., Shang Z., Wanga M. (2020). The sterol carrier hydroxypropyl-β-cyclodextrin enhances the metabolism of phytosterols by *Mycobacterium neoaurum*. Appl. Environ. Microbiol..

[105] Gulla V., Banerjee T., Patil S. (2010). Bioconversion of soysterols to androstenedione by *Mycobacterium fortuitum* subsp. fortuitum NCIM 5239, a mutant derived from total sterol degrader strain. J. Chem. Technol. Biotechnol..

[106] Su L., Shen Y., Gao T., Luo J., Wang M. (2017). Improvement of AD biosynthesis response to enhanced oxygen transfer by oxygen vectors in *Mycobacterium neoaurum* TCCC 11979. Appl. Biochem. Biotechnol..

[107] Nanou K., Roukas T. (2016). Waste cooking oil: A new substrate for carotene production by *Blakeslea trispora* in submerged fermentation. Bioresour. Technol..

[108] Kourmentza C., Costa J., Azevedo Z., Servin C., Grandfils C., De Freitas V., Reis M. (2018). *Burkholderia thailandensis* as a microbial cell factory for the bioconversion of used cooking oil to polyhydroxyalkanoates and rhamnolipids. Bioresour. Technol..

[109] Li H.-X., Lu Z.-M., Geng Y., Gong J.-S., Zhang X.-J., Shi J.-S., Xu Z.-H., Ma Y.-H. (2015). Efficient production of bioactive metabolites from *Antrodia camphorata* ATCC 200183 by asexual reproduction-based repeated batch fermentation. Bioresour. Technol..

[110] Reddy L.V., Kim Y.M., Yun J.S., Ryu H.W., Wee Y.J. (2016). L-Lactic acid production by combined utilization of agricultural bi-oresources as renewable and economical substrates through batch and repeated-batch fermentation of *Enterococcus faecalis* RKY1. Bioresour. Technol..

[111] Han X., Song W., Liu G., Li Z., Yang P., Qu Y. (2017). Improving cellulase productivity of *Penicillium oxalicum* RE-10 by repeated fed-batch fermentation strategy. Bioresour. Technol..

[112] Wijaya H., Sasaki K., Kahar P., Yopi H., Kawaguchi T., Sazuka C., Ogino B., Prasetya A. (2018). Kondo, repeated ethanol fer-mentation from membrane-concentrated sweet sorghum juice using the flocculating yeast Saccharomyces cerevisiae F118 strain. Bioresour. Technol..

[113] Zhang Y., Feng X., Xu H., Yao Z., Ouyang P. (2010). ε-poly-L-lysine production by immobilized cells of *Kitasatospora* sp. MY 5-36 in repeated fed-batch cultures. Bioresour. Technol..

[114] Tang R., Shen Y., Xia M., Tu L., Luo J., Geng Y., Gao T., Zhou H., Zhao Y., Wang M. (2019). A highly efficient step-wise biotransformation strategy for direct conversion of phytosterol to boldenone. Bioresour. Technol..

[115] Chacón S.J., Matias G., Ezeji T.C., Maciel Filho R., Mariano A.P. (2021). Three-stage repeated-batch immobilized cell fermentation to produce butanol from non-detoxified sugarcane bagasse hemicellulose hydrolysates. Bioresour. Technol..

[116] Xu X., Gao X., Feng J., Wang X., Wei D. (2015). Influence of temperature on nucleus degradation of 4-androstene-3, 17-dione in phytosterol biotransformation by *Mycobacterium* sp. Lett. Appl. Microbiol..

[117] Wang X., Hua C., Xu X., Wei D. (2019). Two-step bioprocess for reducing nucleus degradation in phytosterol bioconversion by *Mycobacterium neoaurum* NwIB-R10(hsd4A). Appl. Biochem. Biotechnol..

[118] Bin L.X., Liu H.H., Xu L.Q., Wei D.Z., Wang F.Q. (2017). Role identification and application of sigD in the transformation of soybean phytosterol to 9α-hydroxy-4-androstene-3,17-dione in *Mycobacterium neoaurum*. J. Agric. Food Chem..

[119] Liu K., Gao Y., Li Z.-H., Liu M., Wang F.-Q., Wei D.-Z. (2022). CRISPR-Cas12a assisted precise genome editing of *Mycolicibacterium neoaurum*. New Biotechnol..

[120] Olivera E.R., Luengo J.M. (2019). Steroids as environmental compounds recalcitrant to degradation: Genetic mechanisms of bacterial biodegradation pathways. Genes.

